# Breaking the hierarchy - a new cluster selection mechanism for hierarchical clustering methods

**DOI:** 10.1186/1748-7188-4-12

**Published:** 2009-10-19

**Authors:** László A Zahoránszky, Gyula Y Katona, Péter Hári, András Málnási-Csizmadia, Katharina A Zweig, Gergely Zahoránszky-Köhalmi

**Affiliations:** 1 Department of Computer Science and Information Theory, Budapest University of Technology and Economics, Budapest, Hungary; 2 DELTA Informatika Zrt, Budapest, Hungary; 3 Department of Biochemistry, Eötvös Loránd University, Budapest, Hungary; 4 Department of Biological Physics, Eötvös Loránd University, Budapest, Hungary

## Abstract

**Background:**

Hierarchical clustering methods like Ward's method have been used since decades to understand biological and chemical data sets. In order to get a partition of the data set, it is necessary to choose an optimal level of the hierarchy by a so-called level selection algorithm. In 2005, a new kind of hierarchical clustering method was introduced by Palla et al. that differs in two ways from Ward's method: it can be used on data on which no full similarity matrix is defined and it can produce overlapping clusters, i.e., allow for multiple membership of items in clusters. These features are optimal for biological and chemical data sets but until now no level selection algorithm has been published for this method.

**Results:**

In this article we provide a general selection scheme, the *level independent clustering selection method*, called LInCS. With it, clusters can be selected from any level in quadratic time with respect to the number of clusters. Since hierarchically clustered data is not necessarily associated with a similarity measure, the selection is based on a graph theoretic notion of *cohesive clusters*. We present results of our method on two data sets, a set of drug like molecules and set of protein-protein interaction (PPI) data. In both cases the method provides a clustering with very good sensitivity and specificity values according to a given reference clustering. Moreover, we can show for the PPI data set that our graph theoretic cohesiveness measure indeed chooses biologically homogeneous clusters and disregards inhomogeneous ones in most cases. We finally discuss how the method can be generalized to other hierarchical clustering methods to allow for a level independent cluster selection.

**Conclusion:**

Using our new cluster selection method together with the method by Palla et al. provides a new interesting clustering mechanism that allows to compute overlapping clusters, which is especially valuable for biological and chemical data sets.

## Background

Clustering techniques have been used for decades to find entities that share common properties. Regarding the huge data sets available today, which contain thousands of chemical and biochemical molecules, clustering methods can help to categorize and classify these tremendous amounts of data [1-3]. In the special case of drug design their importance is reflected in their wide-range application from drug discovery to lead molecule optimization [4]. Since structural information of molecules is easier to obtain than their biological activity, the main idea behind using clustering algorithms is to find groups of structurally similar molecules in the hope that they also exhibit the same biological activity. Therefore, clustering of drug-like molecules is a great help to reduce the search space of unknown biologically active compounds.

Several methods that intend to locate clusters have been developed so far. The methods that are used most in chemistry and biochemistry related research are Ward's hierarchical clustering [5], single linkage, complete linkage and group average methods [6]. All of them build hierarchies of clusters, i.e., on the first level of the hierarchy all molecules are seen as similar to each other, but further down the hierarchy, the clusters get more and more specific. To find one single partition of the data set into clusters, it is necessary to determine a level that then determines the number and size of the resultant clusters, e.g., by using the Kelley-index [7]. Note that a too high level will most often lead to a small number of large, unspecific clusters, and that a too low level will on the other hand lead to more specific but maybe very small and too many clusters. A cluster that contains pairwise very similar entities can be said to be *cohesive*. Thus, a level selection algorithm tries to find a level with not too many clusters that are already sufficiently specific or *cohesive*.

Other commonly used clustering methods in chemistry and biology are not based on hierarchies, like the *K*-means [8] and the Jarvis-Patrick method [9]. Note however, that all of the methods mentioned so far rely on a total similarity matrix, i.e., on total information about the data set which might not always be obtainable.

A group of clustering techniques which is not yet so much applied in the field of bio- and chemoinformatics is based on graph theory. Here, molecules are represented by nodes and any kind of similarity relation is represented by an edge between two nodes. The big advantage of graph based clustering lies in those cases where no quantifiable similarity relation is given between the elements of the data set but only a binary relation. This is the case, e.g., for protein-protein-interaction data where the interaction itself is easy to detect but its strength is difficult to quantify; another example are metabolic networks that display whether or not a substrate is transformed into another one by means of an enzyme. The most well-known examples of graph based clustering methods were proposed by Girvan and Newman [10] and Palla et al [11].

The latter method, the so-called *k-clique community clustering (CCC)*, which was also independently described in [12,13], is especially interesting since it cannot only work with incomplete data on biological networks but is also able to produce overlapping clusters. This means that any of the entities in the network can be a member of more than one cluster in the end. This is often a natural assumption in biological and chemical data sets:

1. proteins often have many domains, i.e., many different functions. If a set of proteins is clustered by their function, it is natural to require that some of them should be members of more than one group;

2. similarly, drugs may have more than one target in the body. Clustering in this dimension should thus also allow for multiple membership;

3. molecules can carry more than one active group, i.e., pharmacophore, or one characteristic structural feature like heteroaromatic ring systems. Clustering them by their functional substructures should again allow for overlapping clusters.

This newly proposed method by Palla et al. has already been proven useful in the clustering of *Saccharomyces cerevisiae *[11,14] and human protein-protein-interactions networks [15]. To get a valid clustering of the nodes, it is again necessary to select some level *k*, as for other hierarchical clustering methods. For the CCC the problem of selecting the best level is even worse than in the classic hierarchical clustering methods cited above: while Ward's and other hierarchical clustering methods will only join two clusters per level and thus monotonically decrease the number of clusters from level to level, the number of clusters in the CCC may vary wildly over the levels without any monotonicity as we will show in *'Palla et al.'s clustering method'*.

This work proposes a new way to cut a hierarchy to find the best suitable cluster for each element of the data set. Moreover, our method, the *level-independent cluster selection *or **LInCS **for short does not choose a certain *level *which is optimal but picks the best clusters from all levels, thus allowing for more choices. To introduce LInCS and prove its performance, section *'Methods: the LInCS algorithm' *provides the necessary definitions and a description of the new algorithmic approach. Section *'Data sets and experimental results' *describes the data and section *'Results and discussion' *the experimental results that reveal the potential of the new method. Finally, we generalize the approach in section *'Generalization of the approach' *and conclude with a summary and some future research problems in section *'Conclusions'*.

## Methods: the LInCS algorithm

In this section we first present a set of necessary definitions from graph theory in *'Graph theoretical definitions' *and give a general definition of hierarchical clustering with special emphasis on the CCC method by Palla et al. in *'Hierarchical clustering and the level selection problem'*. Then we introduce the new hierarchy cutting algorithm called *LInCS *in *'Finding cohesive k-clique communities: LInCS'*.

### Graph theoretical definitions

Before we start with sketching the underlying CCC algorithm by Palla et al. and our improvement, the LInCS method, we describe the necessary graph-based definitions.

An *undirected graph G *= (*V*, *E*) consists of a set *V *of nodes, and a set of edges *E *⊆ *V *× *V *that describes a *relation *between the nodes. If {*v*_*i*_, *v*_*j*_} ∈ *E *then *v*_*i *_and *v*_*j *_are said to be *connected *with each other. Note that (*v*_*i*_, *v*_*j*_) will be used to denote an undirected edge between *v *and *w*. The *degree deg*(*v*) of a node *v *is given by the number of edges it is contained in. A *path P(v, w) *is an ordered set of nodes *v *= *v*_0_, *v*_1_,..., *v*_*k *_= *w *such that for any two subsequent nodes in that order (*v*_*i*_, *v*_*i*+1_) is an edge in *E*. The *length of a path *in an unweighted graph is given by the number of edges in it. The *distance d*(*v, w*) between two nodes *v, w *is defined as the minimal length of any path between them. If there is no such path, it is defined to be ∞. A *graph *is said to be connected if all pairs of nodes have a finite distance to each other, i.e., if there exists a path between any two nodes.

A graph *G' *= (*V'*, *E'*) is a *subgraph *of *G *= (*V*, *E*) if *V' *⊆ *V*, *E' *⊆ *E *and *E' *⊆ *V' *× *V'*. In this case we write *G' *≤ *G*. If moreover *V' *≠ *V *then *G' *is a *proper subgraph*, denoted by *G' *<*G*. Any subgraph of *G *that is connected and is not a proper subgraph of a larger, connected subgraph, is called a *connected component of G*.

**Figure 1:**
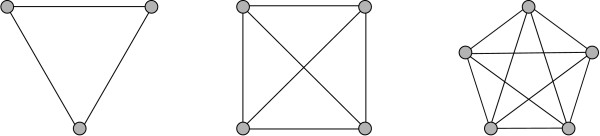
**Shown are a *K*_3_, a *K*_4 _and a *K*_5_**. Note that the *K*_4 _contains 4 *K*_3_, and that the *K*_5 _contains 5 *K*_4 _and 10 *K*_3 _cliques.

A *k-clique *is any (sub-)graph consisting of *k *nodes where each node is connected to every other node. A *k*-clique is denoted by *K*_*k*_. If a subgraph *G' *constitutes a *k*-clique and *G' *is no proper subgraph of a larger clique, it is called a *maximal clique*. Fig. 1 shows examples of a *K*_3_, a *K*_4_, and a *K*_5_.

**Figure 2:**
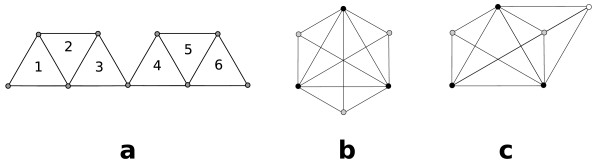
**(a) The *K*_3_s marked by 1 and 2 share two nodes, as do the *K*_3_s marked by 2 and 3, 4 and 5, and 5 and 6**. Each of these pairs is thus 3-adjacent by definition 1. Since 1 and 2 and 2 and 3 are 3-adjacent, 1 and 3 are 3-clique-connected by definition 2. But since 3 and 4 share only one vertex, they are not 3-adjacent. (b) Each of the grey nodes constitutes a *K*_4 _together with the three black nodes. Thus, all three *K*_4_s are 4-adjacent. (c) An example of three *K*_4_s that are 4-clique-connected.

We need the following two definitions given by Palla et al. [11]. See Fig. 2 for examples:

**Definition 1 ***A k-clique A is k-adjacent with k-clique B if they have at least k - 1 nodes in common*.

**Definition 2 ***Two k-cliques C*_1 _*and C_*s *_are k-clique-connected to each other if there is a sequence of k-cliques C*_1_, *C*_2_,..., *C*_*s*-1_, *C*_*s *_*such that C_*i *_and C*_*i*+1 _*are k-adjacent for each i *= 1,..., *s *- 1.

**Figure 3:**
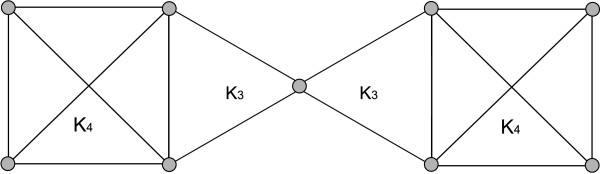
**For *k *= 2, the whole graph builds one 2-clique community, because each edge is a 2-clique, and the graph is connected**. For *k *= 3, there are two 3-clique communities, one consisting of the left hand *K*_4 _and *K*_3_, the other consisting of the right hand *K*_3 _and *K*_4_. The node in the middle of the graph is contained in both 3-node communities. For *k *= 4, each of the *K*_4_s builds one 4-clique community.

This relation is *reflexive*, i.e., clique *A *is always *k*-clique-connected to itself by definition. It is also *symmetric*, i.e., if clique *B *is *k*-clique-connected to clique *A *then *A *is also *k*-clique-connected to *B*. In addition, the relation is *transitive *since if clique *A *is *k*-clique-connected to clique *B *and clique *B *is *k*-clique-connected to *C *then *A *is *k*-clique-connected to *C*. Because the relation is reflexive, symmetric and transitive it belongs to the class of *equivalence relations*. Thus this relation defines *equivalence classes *on the set of *k*-cliques, i.e., there are unique maximal subsets of *k*-cliques that are all *k*-clique-connected to each other. A *k-clique community *is defined as the set of all *k*-cliques in an equivalence class [11]. Fig. 2(a), (b) and 2(c) give examples of *k*-clique communities. A *k-node cluster *is defined as the union of all *nodes *in the cliques of a *k*-clique community. Note that a node can be member of more than one *k*-clique and thus it can be a member of more than *k*-node cluster, as shown in Fig. 3. This explains how the method produces overlapping clusters.

We will make use of the following observations that were already established by Palla et al. [11]:

**Observation 1 ***Let A and B be two cliques of at least size k that share at least k *- 1 *nodes. It is clear that A contains **cliques of size k and B contains **cliques of size k. Note that all of these cliques in A and B are k-clique-connected. Thus, we can generalize the notion of k-adjacency and k-clique-connectedness to cliques of size at least k and not only to those of strictly size k*.

We want to illustrate this observation by an example. Let *C*_1 _be a clique of size 6 and *C*_2 _a clique of size 8. *C*_1 _and *C*_2 _share 4 nodes, denoted by *v*_1_, *v*_2_, *v*_3_, *v*_4_. Note that within *C*_1 _all possible subsets of 5 nodes build a 5-clique. It is easy to see that all of them are 5-clique-connected by definition 1 and 2. The same is true for all possible 5-cliques in *C*_2_. Furthermore, there is at least one 5-clique in *C*_1 _and one in *C*_2 _that share the nodes *v*_1_, *v*_2_, *v*_3_, *v*_4_. Thus, by the transitivity of the relation as given in definition 2, all 5-cliques in *C*_1 _are *k*-clique-connected to all 5-cliques in *C*_2_.

**Observation 2 ***Let C ⊂ C' be a k-clique that is a subset of another clique then C is obviously k-clique-connected to C'. Let C' be k-clique-connected to some clique then due to the transitivity of the relation, C is also k-clique-connected to B. Thus, it suffices to restrict the set of cliques of at least size k to all maximal cliques of at least size k*.

As an illustrative example, let *C*_1 _denote a 4-clique within a 6-clique *C*_2_. *C*_1 _is 4-clique-connected to *C*_2 _because they share any possible subset of 3 nodes out of *C*_1_. If now *C*_2 _shares another 3 nodes with a different clique *C*_3_, by the transitivity of the k-clique-connectedness relation, *C*_1 _and *C*_3 _are also 3-clique-connected. With these graph theoretic notions we will now describe the idea of hierarchical clustering.

### Hierarchical clustering and the level selection problem

A hierarchical clustering method is a special case of a clustering method. A general clustering method produces non-overlapping clusters that build a *partition *of the given set of entities, i.e., a set of subsets such that each entity is contained in exactly one subset. An ideal clustering partitions the set of entities into a *small *number of subsets such that each subset contains *only very similar entities*. Measuring the quality of a clustering is done by a large set of clustering measures, for an overview see, e.g., [16]. If a good clustering can be found, each of the subsets can be meaningfully represented by some member of the set leading to a considerable data reduction or new insights into the structure of the data. With this sketch of general clustering methods, we will now introduce the notion of a hierarchical clustering.

#### Hierarchical clusterings

**Figure 4:**
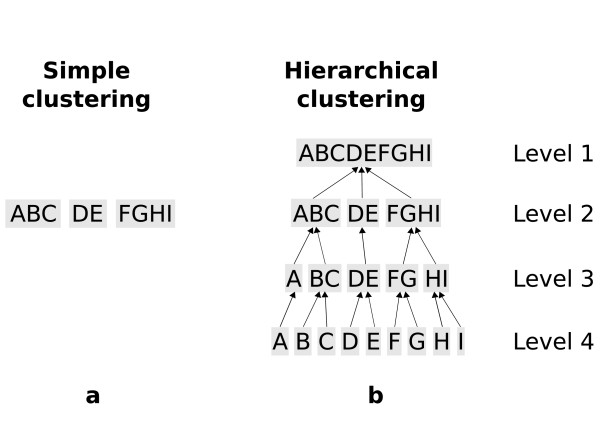
**(a) A simple clustering provides exactly one partition of the given set of entities**. b) A hierarchical clustering method provides many partitions, each associated with a level. The lowest level number is normally associated with the whole data set, and each higher level provides a *refinement *of the lower level. Often, the highest level contains the partition consisting of all *singletons*, i.e., the single elements of the data set.

The elements of a partition *P *= {*S*_1_, *S*_2_,..., *S*_*k*_} are called *clusters *(s. Fig. 4(a)). A hierarchical clustering method produces a set of partitions on different levels 1,..., *k *with the following properties: Let the partition of level 1 be just the given set of entities. A *refinement *of a partition *P *= {*S*_1_, *S*_2_,..., *S*_*j*_} is a partition  such that each element of *P' *is contained in exactly one of the elements of *P*. This containment relation can be depicted as a tree or *dendogramm *(s. Fig. 4(b)).

The most common hierarchical clustering methods start at the bottom of the hierarchy with each entity in its own cluster, building the so-called *singletons*. These methods require the provision of a pairwise distance measure, often called *similarity measure*, of all entities. From this a distance between any two clusters is computed, e.g., the minimum or maximum distance between any two members of the clusters, resulting in single-linkage and complete-linkage clustering [6]. In every step, the two clusters *S*_*i*_, *S*_*j *_with minimal distance are merged into a new cluster. Thus, the partition of the next higher level consists of nearly the same clusters minus *S*_*i*_, *S*_*j *_and plus the newly merged cluster *S*_*i *_∪ *S*_*j*_.

Since a hierarchical clustering computes a set of partitions but a clustering consists of only one partition, it is necessary to determine a level that defines the final partition. This is sometimes called the *k-level selection problem*. Of course, the optimization goals for the optimal clustering are somewhat contradicting: on the one hand, a small number of clusters is wanted. This favors a clustering with only a few large clusters within which not all entities might be very similar to each other. But if, on the other hand, only subsets of entities with high pairwise similarity are allowed, this might result in too many different maximal clusters which does not allow for a high data reduction. Several level selection methods have been proposed to solve this problem so far; the best method for most purposes seems to be the Kelley-index [7], as evaluated by [3]. To find clusters with high inward similarity Kelley et al. measure the average pairwise distance of all entities in one set. Then they create a penalty score out of this value and the number of clusters on every level. They suggest to select the level at which this penalty score is lowest.

We will now shortly sketch Palla et al.'s clustering method, show why it can be considered a hierarchic clustering method although it produces overlapping clusters and work out why Kelley's index cannot be used here to decide the level selection problem.

#### Palla et al.'s clustering method

**Figure 5:**
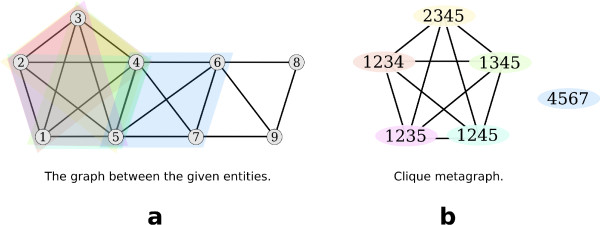
**(a) In the entity-relationship graph the differently colored shapes indicate the different maximal cliques of size 4**. (b) In the clique metagraph every clique is presented by one node and two nodes are connected if the corresponding cliques share at least 3 nodes. Note that nodes 4 and 5 end up in two different node clusters.

Recently, Palla et al. proposed a graph based clustering method that is capable of computing overlapping clusters [11,17,18]. This method has already been proven to be useful, especially in biological networks like protein-protein-interaction networks [14,15]. It needs an input parameter *k *between 1 and the number of nodes *n *with which the algorithm computes the clustering as follows: for any *k *between 1 and *n *compute all maximal cliques of size at least *k*. From this a meta-graph can be built: Represent the maximal cliques as nodes and connect any two of them if they share at least *k *-1 nodes (s. Fig. 5). These cliques are obviously *k*-clique-connected by observations 1 and 2. Any path in the meta-graph connects by definition cliques that are *k*-clique-connected. Thus, a simple connected component analysis in the meta-graph is enough to find all *k*-clique communities. From this, the clusters on the level of the original entities can be easily constructed by merging the entities of all cliques within a *k*-clique community. Note that on the level of the maximal cliques the algorithm constructs a *partition*, i.e., each maximal clique can only be in one *k*-clique community. Since a node can be in different maximal cliques (as illustrated in Fig. 5 for nodes 4 and 5) it can end up in as many different clusters on the *k*-node cluster level.

Note that for *k *= 2 the 2-clique communities are just the connected components of the graph without isolated nodes. Note also that the *k*-clique communities for some level *k *do not necessarily cover all nodes but only those that take part in at least one *k*-clique. To guarantee that all nodes are in at least one cluster, those that are not contained in at least one *k*-node cluster are added as singletons.

We will now show that the *k*-clique communities on different *k*-levels can be considered to build a hierarchy with respect to the containment relation. We will first show a more general theorem and then relate it to the build-up of a hierarchy.

**Theorem 3 ***If k *>*k' *≥ 3 *and two nodes v*, *u are in the same k-node cluster, then there is a k'*-*node cluster containing both u and v*.

This theorem states that if two nodes *u*, *v *are contained in cliques that belong to some *k*-clique community, then, for every smaller *k' *until 3, there will also be a *k'*-clique community that contains cliques containing *u *and *v*. As an example: if *C*_1 _and *C*_2 _are 6-clique-connected, then they are also 5-, 4-, and 3-clique-connected.

**Proof**: By definition 2 *u *and *v *are in the same *k*-clique community if there is a sequence of *k*-cliques *C*_1_, *C*_2_,..., *C*_*s*-1_, *C*_*s *_such that *C*_*i *_and *C*_*i*+1 _are *k*-adjacent for each *i *= 1,..., s -1, and such that *u *∈ *C*_1_, *v *∈ *C*_*s*_. In other words, there is a sequence of nodes *u *= *v*_1_, *v*_2_,..., *v*_*s*+*k*-1 _= *v*, such that *v*_*i*_, *v*_*i*+1_,..., *v*_*i*+*k*-1 _is a *k*-clique for each 1 ≤ *i *≤ *s*.

It is easy to see that in this case the subset of nodes *v*_*i*_, *v*_*i*+1_,..., *v*_*i*+*k'*-1 _constitutes a *k'*-clique for each 1 ≤ *i *≤ *s *+ *k *- *k'*. Thus by definition there is a *k'*-clique community that contains both *u *and *v*.   ■

**Figure 6:**
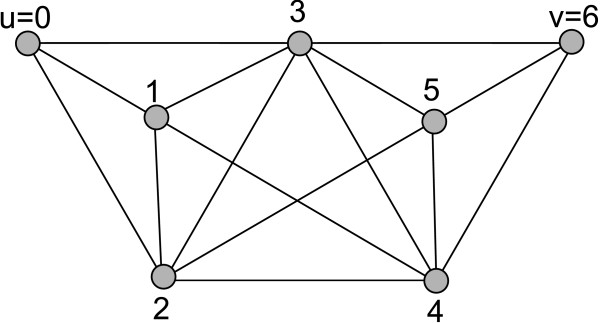
***u *= 0 and *v *= 6 are in cliques that are 4-clique-connected because clique (0, 1, 2, 3) is 4-clique adjacent to clique (1, 2, 3, 4), which is in turn 4-clique-adjacent to clique (2, 3, 4, 5), which is 4-clique-adjacent to clique (3, 4, 5, 6)**. It is also easy to see that every three consecutive nodes build a 3-clique and that two subsequent 3-cliques are 3-clique-adjacent, as stated in Theorem 3. Thus, *u *and *v *are contained in cliques that are 3-clique-connected.

The proof is illustrated in Fig. 6. Moreover the theorem shows that if two cliques are *k*-clique connected, they are also *k'*-clique connected for each *k *>*k' *≥ 3. This general theorem is of course also true for the special case of *k' *= *k *- 1, i.e., if two cliques are in a *k*-clique community, they are also in at least one *k *- 1-clique community. We will now show that they are only contained in at most one *k *- 1-clique community:

**Theorem 4 ***Let the different k-clique communities be represented by nodes and connect node A and node B by a directed edge from A to B if the corresponding k-clique community C*_*A *_*of A is on level k and B's corresponding community C*_*B *_*is on level k *- 1 *and C*_*A *_*is a subset of or equal to C*_*B*_. *The resulting graph will consist of one or more trees, i.e., the k-clique communities are hierarchic with respect to the containment relation*.

**Proof**: By Theorem 3 each *k*-clique community with *k *> 3 is contained in at least one *k *-1-clique community. Due to the transitivity of the *k*-connectedness relation, there can be only one *k *- 1-clique community that contains any given *k*-clique community. Thus, every *k*-clique community is contained in exactly one *k *- 1-clique community.

There are two important observations to make:   ■

**Figure 7:**
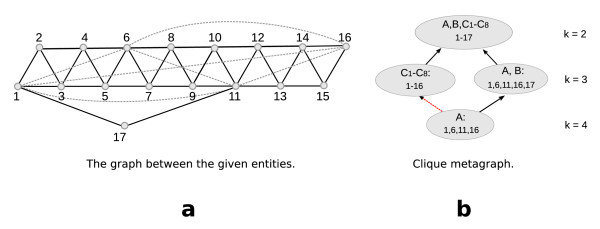
**(a) The example shows one maximal clique A of size 4 with *A *= (1, 6, 11, 16) (dashed, grey lines), and 11 maximal cliques of size 3, namely *B *= (1, 11, 17) and *C*_*i *_= (*i*, *i *+1, *i *+2) for all 1 ≤ *i *≤ 14**. Note that *A *and *B *share two nodes with each other but at most one node with every of the *C*_*i *_cliques. (b) Clique *A *constitutes the only 4-clique community on level 4. On level 3 we see one 3-clique community consisting of all Ci cliques and one consisting of *A *and *B*. Note that, as stated in Theorem 4, clique *A *is contained in only one 3-clique community. However, the set of nodes (1, 6, 11, 16) is contained in **both **of the corresponding 3-node clusters. The containment relation is indicated by the red, dashed arrow. Thus this graph provides an example where the containment relationship on the level of *k*-node clusters does not have to be hierarchic. This graph is additionally an example for a case in which the number of *k*-clique communities is neither monotonically increasing nor decreasing with increasing *k*.

**Observation 3 ***Given the set of all k-node clusters (instead of the k-clique communities) for all k, these could also be connected by the containment relationship. Note however that this will not necessarily lead to a hierarchy, i.e., one k-node cluster can be contained in more than one k *- 1-*node cluster (s. Fig*. 7).

**Observation 4 ***Note also that the number of k-node clusters might neither be monotonically increasing nor decreasing with k (s. Fig*. 7).

It is thus established that on the level of *k*-clique communities, the CCC builds a hierarchical clustering. Of course, since maximal cliques have to be found in order to build the *k*-clique communities, this method can be computationally problematic [19], although in practice it performs very well. In general, CCC is advantageous in the following cases:

1. if the given data set does not allow for a meaningful, real-valued similarity or dissimilarity relationship, defined for all pairs of entities;

2. if it is more natural to assume that clusters of entities might overlap.

It is clear that this clustering method bears the same *k*-level selection problem as other hierarchical clustering methods. Moreover, the number and size of clusters can change strongly from level to level. Obviously, since quantifiable similarity measures might not be given, Kelley's index cannot be used easily. Moreover, it might be more beneficial to select not a whole level, but rather to find for each maximal clique the one *k*-clique community that is at the same time *cohesive *and *maximal*. The next section introduces a new approach to finding such a *k*-clique community for each maximal clique, the *level independent cluster selection mechanism (LInCS)*.

### Finding cohesive *k*-clique communities: LInCS

Typically, at lower values of *k*, e.g., *k *= 3, 4, large clusters are discovered, which tend to contain the majority of entities. This suggests a low level of similarity between some of them. Conversely, small clusters at larger *k*-values are more likely to show higher level of similarity between all pairs of entities. A cluster in which all pairs of entities are similar to one another will be called a *cohesive cluster*. Note that a high value of *k *might also leave many entities as singletons since they do not take part in any clique of size *k*.

**Figure 8:**
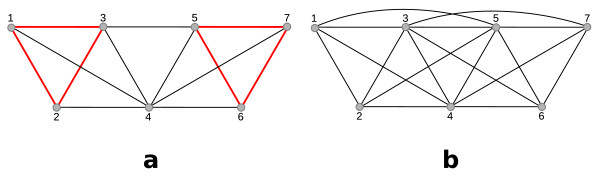
**(a) This graph consists of three maximal cliques: (1, 2, 3, 4), (4, 5, 6), and (4, 5, 6, 7)**. The 3-clique community on level 3 is not cohesive because there are two 3-cliques, namely (1, 2, 3) and (5, 6, 7), indicated by red, bold edges, that do not share a node. An equivalent argumentation is that the union of (1, 2, 3, 4) and (4, 5, 6, 7) contains 7 distinct nodes, i.e., more than 2*k *= 6 nodes. Both 4-clique communities are cohesive because they consist of a single clique with size less than 2*k *= 8. (b) This graph consists of a two maximal cliques: (1, 2, 3, 4, 5) and (3, 4, 5, 6, 7). On both levels, 3 and 4, the *k*-clique community consists of both cliques, but on level 3 the 3-clique community is **not **cohesive because (1, 2, 3) and (5, 6, 7) still share no single node. But on level 4 the 4-clique community is cohesive because the union of the two maximal cliques contains 7, i.e., less than 2*k *= 8 nodes.

Since the CCC is often used on data sets where no meaningful pairwise distance function can be given, the question remains of cohesion within a cluster can be meaningfully defined. It does not seem to be possible on the level of the *k*-node clusters. Instead, we use the level of the *k*-clique communities and define a given *k*-clique community to be *cohesive *if all of its constituting *k*-cliques share at least one node (s. Fig. 8):

**Definition 5 ***A k-clique community satisfies the *strict clique overlap criterion *if any two k-cliques in the k-clique community overlap (i.e., they have a common node). The k-clique community itself is said to be *cohesive.

A *k*-clique community is defined to be *maximally cohesive *if the following definition applies:

**Definition 6 ***A k-clique community is *maximally cohesive *if it is cohesive and there is no other cohesive k-clique community of which it is a proper subset*.

The CCC was implemented by Palla et al., resulting in a software called the *CFinder *[20]. The output of CFinder contains the set of all maximal cliques, the overlap-matrix of cliques, i.e., the number of shared nodes for all pairs of maximal cliques, and the *k*-clique-communities. Given this output of CFinder, we will now show how to compute all maximally cohesive *k*-clique communities.

**Theorem 7 ***A k-clique community is cohesive if and only if it fulfills one of the following properties:*

*1. A k-clique community is cohesive if and only if either it contains only one clique and this contains less than *2*k nodes, or*

*2. if the union of any two cliques K*_*x *_*and K*_*y *_*in the community has less than *2*k nodes. Note that this implies that the number of shared nodes z has to be larger than x *+ *y *- 2*k*.

**Figure 9:**
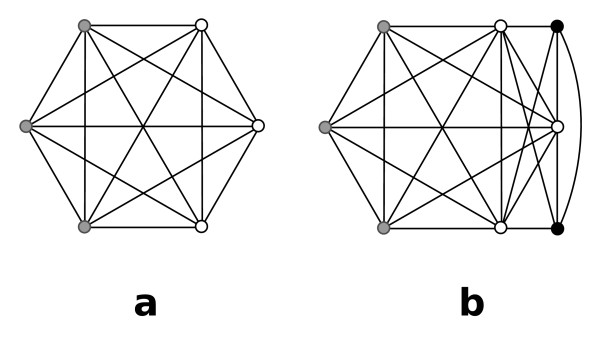
**(a) The *K*_6 _is not cohesive as a 3-clique community because it contains two 3-cliques (indicated by grey and white nodes) that do not share a node**. However, it is a cohesive 4-, 5-, or 6-clique community. (b) The graph constitutes a 3- and a 4-clique community because the *K*_6 _(grey and white nodes) and the *K*_5 _(white and black nodes) share 3-nodes. However, the union of the two cliques contains 8 nodes, and thus it is not cohesive on both levels. For *k *= 3, the grey nodes build a *K*_3_, which does not share a node with the *K*_3 _built by the white nodes; for *k *= 4, the grey nodes and any of the white nodes build a *K*_4_, which does not share any node with the *K*_4 _built by the other 4 nodes.

This theorem states that we can also check the cohesiveness of a *k*-clique community if we do not know all constituting *k*-cliques but only the constituting **maximal **cliques. I.e., the latter can contain more than *k *nodes. Since our definition of cohesiveness is given on the level of *k*-cliques, this new theorem helps to understand its significance on the level of maximal cliques. The proof is illustrated in Fig. 9.

**Proof**: *(1)*: If the *k*-clique community consists of one clique of size ≥ 2*k *then one can find two disjoint cliques of size *k*, contradicting the strict clique overlap criterion. If the clique consists of less than 2*k *nodes it is not possible to find two disjoint cliques of size *k*.

*(2) *Note first that since the *k*-clique community is the union of cliques with at least size *k*, it follows that *x*, *y *≥ *k*. Assume that there are two cliques *K*_*x *_and *K*_*y *_and let *K*_*x*∩*y *_:= *K*_*x *_∩ *K*_*y *_denote the set of shared nodes. Let furthermore |*K*_*x*∩*y*_| = *z*, *K*_*x∪y *_:= *K*_*x *_∪ *K*_*y *_and let their union have at least 2*k *nodes: |*K*_*x*∪*y*_| = *x *+ *y *- *z *≥ 2*k*. It follows that *z *>*x *+ *y *- 2*k*. If now *x *- *z *≥ *k *choose any *k *nodes from *K*_*x*_\*K*_*y *_and any *k *nodes from *K*_*y*_. These two sets constitute *k*-cliques that are naturally disjoint. If *x *-*z *<*k *add any *k *-(*x *-*z*) nodes from *K*_*x*∩*y*_, building *k*-clique *C*_1_. Naturally, *K*_*y*_\*C*_1 _will contain at least *y *- (*k *- *x *+ *z*)) = *y *- *k *+ *x *- *z *>*k *nodes. Pick any *k *nodes from this to build the second *k*-clique *C*_2_. *C*_1 _and *C*_2 _are again disjoint. It thus follows that if the union of two cliques contains at least 2*k *nodes, one can find two disjoint cliques of size *k *in them. If the union of the two cliques contains less than 2*k *distinct nodes it is not possible to find two sets of size *k *that do not share a common node which completes the proof.   ■

With this, a simple algorithm to find all cohesive *k*-clique communities is given by checking for each *k*-clique community on each level *k *first whether it is cohesive:

1. Check whether any of its constituting maximal cliques has a size larger than 2*k *- then it is not cohesive. This can be done in *O*(1) in an appropriate data structure of the *k*-clique communities, e.g., if stored as a list of cliques. Let *γ *denote the number of maximal cliques in the graph. Since every maximal clique is contained in at most one *k*-clique community on each level, this amounts to *O*(*k*_*max*_*γ*).

2. Check for every pair of cliques *K*_*x*_, *K*_*y *_in it whether their overlap is larger than *x *+ *y *- 2*k *- then it is not cohesive. Again, since every clique can be contained in at most one *k*-clique community on each level, this amounts to *O*(*k*_*max*_*γ*^2^).

The more challenging task is to prove maximality. In a naive approach, every of the *k*-clique communities has to be checked against all other *k*-clique communities whether it is a subset of any of these. Since there are at most *k*_*max*_*γ *many *k*-clique communities with each at most *γ *many cliques contained in them, this approach results in a runtime of . Luckily this can be improved to the following runtime:

**Theorem 8 ***To find all maximally cohesive k-clique communities given the clique-clique overlap matrix M takes O*(*k*_*max *_· *γ*^2^).

The proof can be found in the Appendix.

Of course, *γ *can in the worst case be an exponential number [19]. However, CFinder has proven itself to be very useful in the analysis of very large data sets with up to 10, 000 nodes [21]. Real-world networks neither tend to have a large *k*_*max *_nor a large number of different maximal cliques. Thus, although the runtime seems to be quite prohibitive it turns out that for the data sets that show up in biological and chemical fields the algorithm behaves nicely. Of course, there are several other algorithms for computing the set of all maximal cliques, especially on special graph classes, like sparse graphs or graphs with a limited number of cliques. A good survey on these algorithm can be found in [22]. The algorithm in [23] runs in *O*(*nmγ*), with *n *the number of nodes and *m *the number of edges in the original graph. Determining the clique-clique overlap matrix takes *O*(*nγ*^2^) time, and with this we come to an overall runtime of *O*(*n*(*mγ *+ *γ*^2^)). Computing the *k*-clique communities for a given *k*, starting from 3 and increasing it, can be done by first setting all entries smaller than *k *- 1 to 0. Under the reasonable assumption that the number of different maximal cliques in real-world networks can be bound by a polynomial the whole runtime is polynomial.

*Algorithm ***LInCS**

Input: Clique-Clique-Overlap Matrix *M*

**for **k = 3 to *k*_*max *_**do**

   Build graph *G*(*k*) in which two cliques *C*_*i*_, *C*_*j *_are connected by an

   edge if *M *[*i*] [*j*] ≥ *k *- 1

   (*k*) ← compute components in *G*(*k*)

   **for all **components *C *in (*k*) **do**

      **if **isCohesive(C, M) **then**

         Insert *C *into the list of recognized maximally cohesive *k*-clique communities

         Remove all maximal cliques in *C *from *M*

      **end if**

   **end for**

end for

*Bool function ***isCohesive**

**for **i = 1 to number of cliques in *k*-clique-community *C ***do**

   **if **clique1 has more than 2*k *nodes **then**

      **return **FALSE

   **end if**

end for

**for **all pairs of cliques *C*_*i *_and *C*_*j *_**do**

   **if ***C*_*i *_is a *K*_*x *_clique and *C*_*j *_is a *K*_*y *_clique and M [*i*] [*j*] <*x *+ *y *- 2*k ***then**

      **return **FALSE

   **end if**

end for

**return **TRUE

In the following section we will describe some results on the performance of the LInCS-algorithm on different data sets.

## Data sets and experimental results

In subsection *'Data sets' *we introduce the data sets that were used to evaluate the quality of the new clustering algorithm. Subsection *'Performance measurement of clustering molecules' *describes how to quantify the quality of a clustering of some algorithm with a given reference clustering.

### Data sets

We have applied LInCS to two different data sets: the first data set consists of drug-like molecules and provides a natural clustering into six distinct clusters. Thus, the result of the clustering can be compared with the natural clustering in the data set. Furthermore, since this data set allows for a pairwise similarity measure, it can be compared with the result of a classic Ward clustering with level selection by Kelley. This data set is introduced in *'Drug-like molecules'*. The next data set on protein-protein interactions shows why it is necessary to allow for graph based clustering methods that can moreover compute overlapping clusters.

#### Drug-like molecules

**Figure 10:**
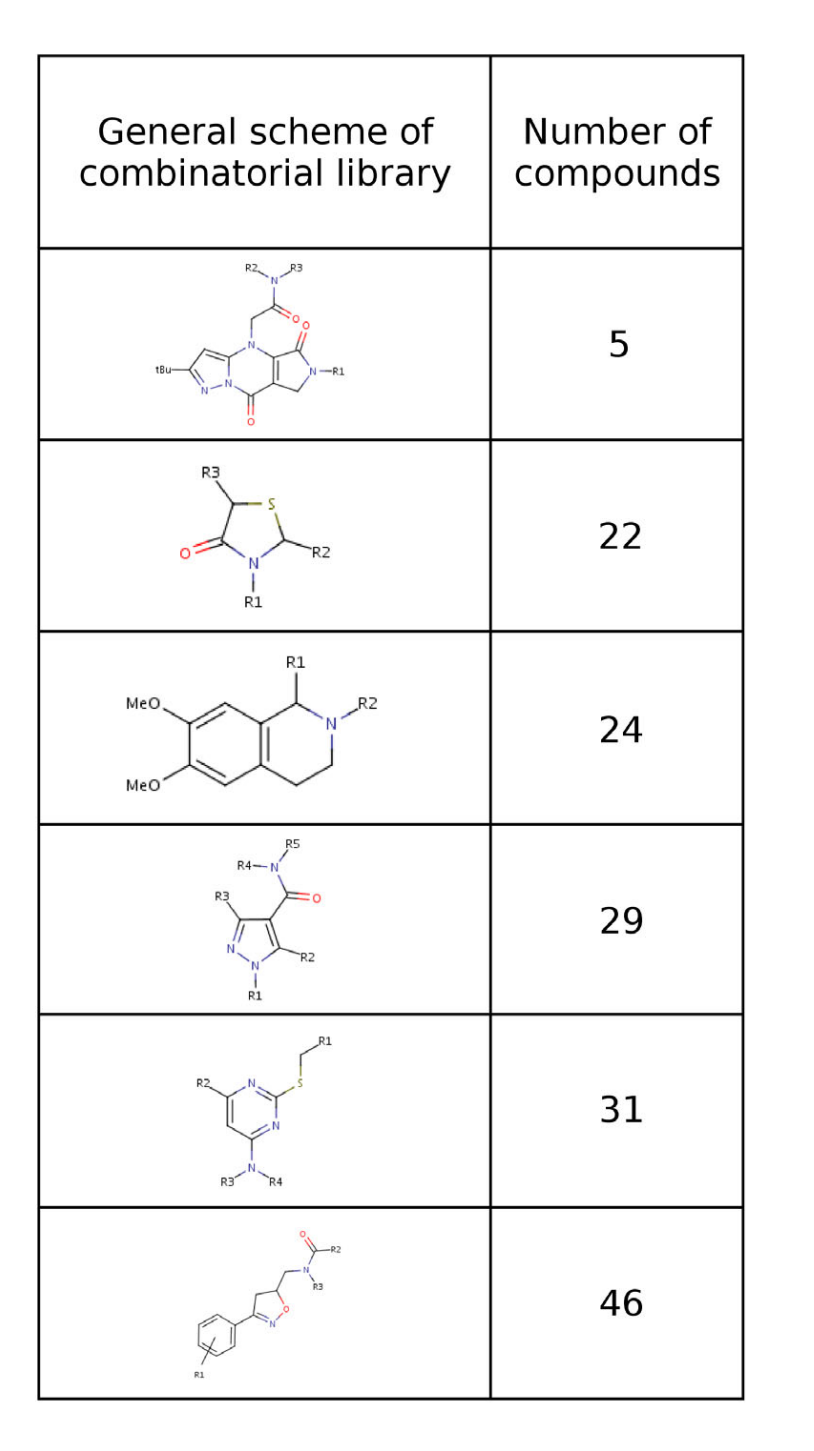
**The first data set consists of drug-like molecules from six different combinatorial libraries**. The figure presents the general structural scheme of molecules in each combinatorial library and the number of compounds in it.

157 drug-like molecules were chosen as a reference data set to evaluate the performance of LInCS with respect to the most used combination of Ward's clustering plus level selection by Kelley et al.'s method. The molecules were downloaded from the ZINC database which contains commercially available drug-like molecules [24]. The chosen molecules belong to six groups that all have the same *scaffold*, i.e., the same basic ring systems, enhanced by different combinations of side chains. The data was provided by the former ComGenex Inc., now Albany Molecular Research Inc. [25]. Thus, within each group, the molecules have a basic structural similarity; the six groups are set as reference clustering. Fig. 10 shows the general structural scheme of each of the six subsets and gives the number of compounds in each library. Table 1, 2 gives the IDs of all 157 molecules with which they can be downloaded from ZINC.

**Table 1:** The table gives the ZINC database IDs for the 157 drug-like molecules that are manually clustered in 6 groups, depending on their basic ring systems (clusters 1 to 3).

**Cluster 1**	**Cluster 2**	**Cluster 3**
06873823	06873893	06873927
06873855	06873894	06873929
06873857	06873895	06874039
06873861	06874719	06874040
06874015	06874720	06874109
06874088	06874722	06874174
06874162	06874724	06874175
06874204	06874725	06874176
06874206	06874726	06874178
06874209	06874727	06874243
06874212	06874728	06874244
06874300	06874729	06874256
06874301	06874732	06874257
06874342	06874733	06874258
06874351	06874734	06874259
06874352	06874750	06874260
06874356	06874764	06874262
06874360	06874767	06874921
06874361	06874768	06874923
06874364	06874769	06874924
06874479	06874771	06874925
06874527	06874772	06874928
06874531	06874789	06875012
06874540	06874790	06875013
06874573	06874792	06875014
06874578	06874793	06875015
06874579	06874794	06875016
06874583	06874795	06875017
06874586	06874802	06875018
06874588	06874912	
06874597	06875068	
06874599		
06874634		
06874635		
06874639		
06874696		
06874833		
06874836		
06874975		
06875048		
06875051		
06875052		
06875055		
06875058		
06875060		
06875064		

To each ID the prefix *ZINC *has to be added, i.e., first molecule's ID is: ZINC06873823.

**Table 2:** The table gives the ZINC database IDs for the 157 drug-like molecules that are manually clustered in 6 groups, depending on their basic ring systems (clusters 4 to 6). To each ID the prefix *ZINC *has to be added.

**Cluster 4**	**Cluster 5**	**Cluster 6**
06874736	06873829	06873904
06874744	06873897	06874113
06874755	06873901	06874115
06874798	06873930	06874117
06874808	06873933	06874119
06874864	06873939	
06874868	06873956	
06874869	06874021	
06874875	06874026	
06874877	06874030	
06874881	06874032	
06874882	06874035	
06874885	06874172	
06874886	06874192	
06874889	06874194	
06874897	06874197	
06875071	06874201	
06875075	06874554	
06875077	06874560	
06875082	06875035	
06875084	06875039	
06875086	06875044	
06875091		
06875097		

As already indicated, this data does not come in the form of a graph or network. But it is easy to define a similarity function for every pair of molecules, as sketched in the following.

### Similarity metric of molecules

The easiest way to determine the similarity of two molecules is to use a so-called 2*D*-fingerprint that encodes the two-dimensional structure of each molecule. 2*D *molecular fingerprints are broadly used to encode 2D structural properties of molecules [26]. Despite their relatively simple, graph-theory based information content, they are also known to be useful for clustering molecules [4].

Although different fingerprinting methods exist, hashed binary fingerprint methods attracted our attention due to their simplicity, computational cost-efficiency and good performance in the case of combinatorial libraries [2,3]. One of the most commonly used hashed fingerprint algorithms is the Daylight-algorithm [27]. A similar algorithm was implemented by the ChemAxon Inc. [28] to produce ChemAxon hashed binary fingerprints.

The binary hashed fingerprint generating procedure first explores all the substructures in the molecule up to a predefined number of bonds (typically 6 bonds). The following step converts each discovered fragment into an integer based on a scoring table. The typical length, i.e., the number of bits of a hashed fingerprint are 1024, 2048, or higher, and initially the value of bits are set to 0. The presence of a discovered fragment in the fingerprint is represented by turning the value of the bit from 0 to 1 at the bit position computed by the score of the fragment. In summary, the fingerprints try to map substructures in a molecule into numbers with the idea that molecules with similar overall structure will have many substructures in common and thus also their fingerprints will be alike.

In this study we used the freely available ChemAxon fingerprint method [28]. Fingerprints for molecules were produced with a length of 4096 bits by exploring up to 6 bonds.

In order to quantify the level of similarity between the fingerprints of two molecules we applied the well-known Tanimoto-similarity coefficient [29]. The Tanimoto-similarity coefficient of molecules *A *and *B *(*T*_*AB*_) is computed according to Eq. 1. In the formula, *c *denotes the number of common bits in the fingerprint of molecule *A *and *B*, and *a *and *b *stand for the number of bits contained by molecule *A *and *B*, respectively. The value of the Tanimoto-similarity coefficient ranges from 0 (least similar) to 1 (most similar).

(1)

The Tanimoto-similarity coefficient of each pair of molecules can be computed and then stored in a matrix, denoted as the *similarity matrix*. As stated above, the CCC method and LInCS expect as input a graph or network that is not fully connected, otherwise the outcome is just the whole set of entities. In the following we will describe how a reasonable similarity-threshold *t *can be found to turn the similarity matrix into a meaningful adjacency matrix. With this, we will then define a graph by representing all molecules by nodes and connect two nodes if their similarity is higher than that threshold.

### Generating a similarity network of molecules

It is clear that the choice of a threshold value *t *might strongly influence the resulting clustering; thus, a reasonable selection of *t *is very important. To our knowledge, no general solution exists for selecting the optimal *t*. It is clear that if *t *is too high, the resulting graph will have only a few number of edges and will consist of several components. But it can be expected that these components will show a high density of edges and will be almost clique-like. This can actually be measured by the so-called *clustering coefficient *that was introduced by Watts and Strogatz [30]. The clustering coefficient of a single node *v *computes the ratio between the number *e*(*v*) of its neighbors that are directly connected to each other and the possible number of neighbors that could be connected to each other, given by *deg*(*v*)·(*deg*(*v*) - 1)/2:

(2)

The clustering coefficient of a graph is defined as the average clustering coefficient of its nodes. Starting at *t *= 1, the resulting graph is empty. By decreasing *t*, more and more edges will be introduced, connecting the isolated components with each other and thus increasing the average clustering coefficient.

**Figure 11:**
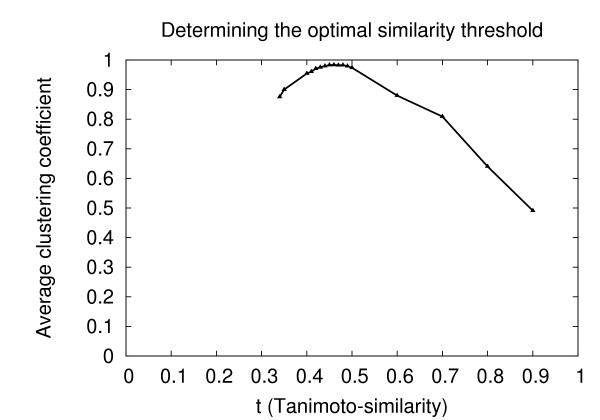
**Average clustering coefficient vs. similarity threshold**. The diagram shows a clear local maximum at *t *= 0.46 where the average clustering coefficient is 0.9834.

Below a certain *t*, it is likely that the new edge will be connecting more or less random pairs of nodes and thus decrease the clustering coefficient of the newly connected nodes. Of course, if the threshold value is set to 0, the resulting graph would be one big clique and the clustering coefficient would again rise to 1. Based on this general behavior, the question is whether there is a natural choice for *t *where the clustering coefficient is still high and *t *is not yet too low. To understand the dependency between the average clustering coefficient and t, we computed all graphs with thresholds *t *ranging from 0.34--which is the average similarity between all molecules in the set--to 1. Surprisingly, there is a local maximum at *t *= 0.46 with an average clustering coefficient of 0.9834 (s. Fig. 11). This value was then chosen as the optimal threshold value.

On this data set we have applied Ward's hierarchical clustering with a level selection according to Kelley's index and compared it with the reference clustering and the one computed by our LInCS algorithm. The results are discussed in *'Performance of LInCS on the drug-like molecule data set'*. We will now introduce the second data set that does not allow for a simple pairwise similarity measure but is rather described naturally by a graph.

#### Protein-protein interaction network of yeast core

Protein-protein interaction data present a very good example of biological data that does not come with a quantifiable pairwise similarity but with a binary relationship: either two proteins are either observed to interact or not. Thus, classic hierarchical clustering methods cannot be applied here. Furthermore, it is assumed that many proteins may have more than one function in the cell; e.g., in the yeast protein database one or more of 42 *cellular roles *can be assigned to a given protein. According to [31], 39% of the proteins were assigned multiple cellular roles. Here, a clustering method is clearly advantageous if it can produce overlapping clusters.

The data was used as provided by Palla et al. in their CFinder-software package which is available on the Internet [20] under the name ScereCR20050417. In the whole it contains 2, 640 proteins. The data is based on experimental data which are curated manually as well as automatically. Some of the most used protein-protein interaction detection methods, as the yeast two-hybrid system are well-known for the poor quality of their results; some estimate that the number of false-positive results is up to 50% [32]. In this case a clustering method can help to find out those edges that are more likely to be correct. To create a graph, each protein is presented by a node and two nodes are connected by an edge if the corresponding proteins are reported to interact. The resulting graph is unweighted and undirected.

### Performance measurement of clustering molecules

After having introduced the data sets, we will now show how a given clustering's quality can be quantified if some reference clustering is given.

#### Sensitivity and specificity

We will first discuss the case of a non-overlapping reference clustering and then that of an overlapping reference clustering.

Given any non-overlapping reference clustering, as the set of 157 molecules that naturally come in six clusters, the clustering of some algorithm *X *has to be compared with it in a reasonable way. The correctness of the clustering process can be quantified by the so-called *sensitivity *and *specificity *measures [33]. Both rely on the following idea, exemplified on the molecule data set: Let *A*_*CL *_denote a matrix of size 157 × 157 where there is a 1 in *A*_*CL*_[*i*][*j*] if any combinatorial library contained both molecule *m*_*i *_and *m*_*j *_(and a -1 otherwise). Let similarly *A*_*X *_denote the corresponding matrix that results from the clustering by algorithm *X*, i.e., *A*_*X*_[*i*][*j*] contains a 1 if algorithm *X *detects a cluster in which *m*_*i *_and *m*_*j *_are both contained and a -1, otherwise. Since *A*_*CL *_is the standard, an algorithm performs well if it makes the same decisions of whether a pair of molecules is in the same cluster or not. We distinguish the following four cases:

1. *A*_*CL*_[*i*][*j*] = 1 &*A*_*X *_[*i*][*j*] = 1: true positive;

2. *A*_*CL*_[*i*][*j*] = -1 &*A*_*X *_[*i*][*j*] = -1: true negative;

3. *A*_*CL*_[*i*][*j*] = -1 &*A*_*X *_[*i*][*j*] = 1: false positive;

4. *A*_*CL*_[*i*][*j*] = 1 &*A*_*X *_[*i*][*j*] = -1: false negative;

With this, the *sensitivity *is defined as the number of pairs of molecules that are correctly clustered together by algorithm *X *divided by all the pairs that were clustered together in the reference clustering. This latter number can be expressed by the sum of all true positive and false negative pairs as can be easily seen from the enumeration above:

(3)

If the sensitivity is 1, this means that algorithm *X *has clustered together exactly the same pairs of molecules as the reference clustering. However, this would also happen, e.g., if *X *simply produces one big cluster. To rule this out, the *specificity *is defined as the ratio of the number of pairs correctly separated into *different clusters*, divided by the number of pairs that are in different clusters in the reference clustering. This latter number can be analogously represented by the sum of the number of true negatives plus the number of false positives:

(4)

Thus, the clustering computed by some algorithm *X *has a specificity of 1 if and only if it puts all pairs of molecules into different clusters that are in different clusters in the reference clustering. If the clustering computed by some algorithm *X *shows a sensitivity **and **specificity of 1 it has found exactly the same clusters as the reference clustering. Note that for a hierarchical clustering the *sensitivity *of a clustering in level *k *is at least as large as the sensitivity of clusterings in higher levels while the *specificity *is at most as large as that of clusterings in higher levels.

The case is a bit more difficult for overlapping reference clusterings as in the case of the protein-protein-interaction network where no simple reference clustering exists. However, for many proteins annotation data is available that assigns different kind of properties to them, e.g.:

1. the biological processes in which the protein is involved;

2. the cellular component in which the protein is located;

3. the molecular function of the protein.

Using the BINGO 2.0 plugin [34] of Cytoscape [35] it is possible to obtain for a given clustering of proteins all of the annotated functions from the three categories as described above. Actually, these are downloaded from different databases, as described by [34]. One of the possible applications of a clustering algorithm on this kind of data is that proteins that are not yet assigned to, e.g., a biological process might be assigned to a cluster in which almost all proteins are annotated to the same biological process. Since a cluster represents a set of strongly interacting proteins it is then very likely that also the not yet annotated protein takes part in this biological process. This can then be checked directly in the laboratory, possibly saving money and time for other tests. To understand whether the clusterings obtained by the CCC and LInCS method are pure enough for most annotation categories, we analyzed them with respect to all annotation categories. Each category is thought of as a cluster in the reference clustering. Since more than one category can be assigned to each protein, this can be thought of as an overlapping reference clustering. To make the quality measurements meaningful in this case we will have to alter them slightly as described in the following.

Let  denote the set of all proteins and let *P*(*bp*) denote the set of all proteins that are assigned to at least one biological process. This amounts to 2, 198 proteins. Similarly, *P*(*cc*) denotes the subset of all proteins that are assigned to at least one cellular component with |*P*(*cc*)| = 2, 184, and *P*(*mf*) the subset of all proteins of which at least one molecular function is known with |*P*(*mf*)| = 1, 822.

Each single biological process, cellular component, or molecular function will in the following be called a *category*. *C*_*X*_, *X *∈ {*bp*, *mf*, *cc*}, denotes the type of the category: *C*_*bp *_denotes a specific biological process and analogously *C*_*mf *_and *C*_*cc *_a cellular component. Let now (*C*) denote the subset of proteins that is assigned to category *C*. Of course, a clustering is *sensitive *if all proteins in (*C*) are in one cluster.Regarding a single category *C*_*X*_, e.g., a biological process, we will call a clustering *specific *if no protein from (*C*_*X*_) is in a cluster with any protein that is at least assigned to one biological process but not to *C*_*X*_, i.e., any protein in *P *(*X*) - *P*(*C*_*X*_). However, we do not care whether any pair of two proteins from (*P*(*X*) - *P*(*C*_*X*_)) × (*P*(*X*) - *P*(*C*_*X*_)) are together in a cluster or not. This is justified because these proteins in *P*(*X*) - *P*(*C*_*X*_) could be assigned to other categories of the same type that require them to be in the same cluster. This implies that we should not require them to be in separate clusters. We thus restrict the measurement of the *sensitivity *to all pairs in (*C*_*X*_) × (*C*_*X*_) and the *specificity *to all pairs in (*C*_*X*_) × (*P*(*X*) - *P*(*C*_*X*_)).

After describing the data sets and the methods to quantify the result of a given clustering, we will now describe the results.

## Results and discussion

### Performance of LInCS on the drug-like molecule data set

The molecule data set described in *'Drug-like molecules' *is the data set to which all methods can be applied: Ward's hierarchical clustering together with the level-selection mechanism defined by Kelley-index and the two overlapping clustering algorithms CCC and LInCS. We first discuss the clusters resulting from Ward's hierarchical clustering together with the Kelley-index, then discuss the clusters on different k-levels as computed by the CCC and finally show the results for LInCS.

**Figure 12:**
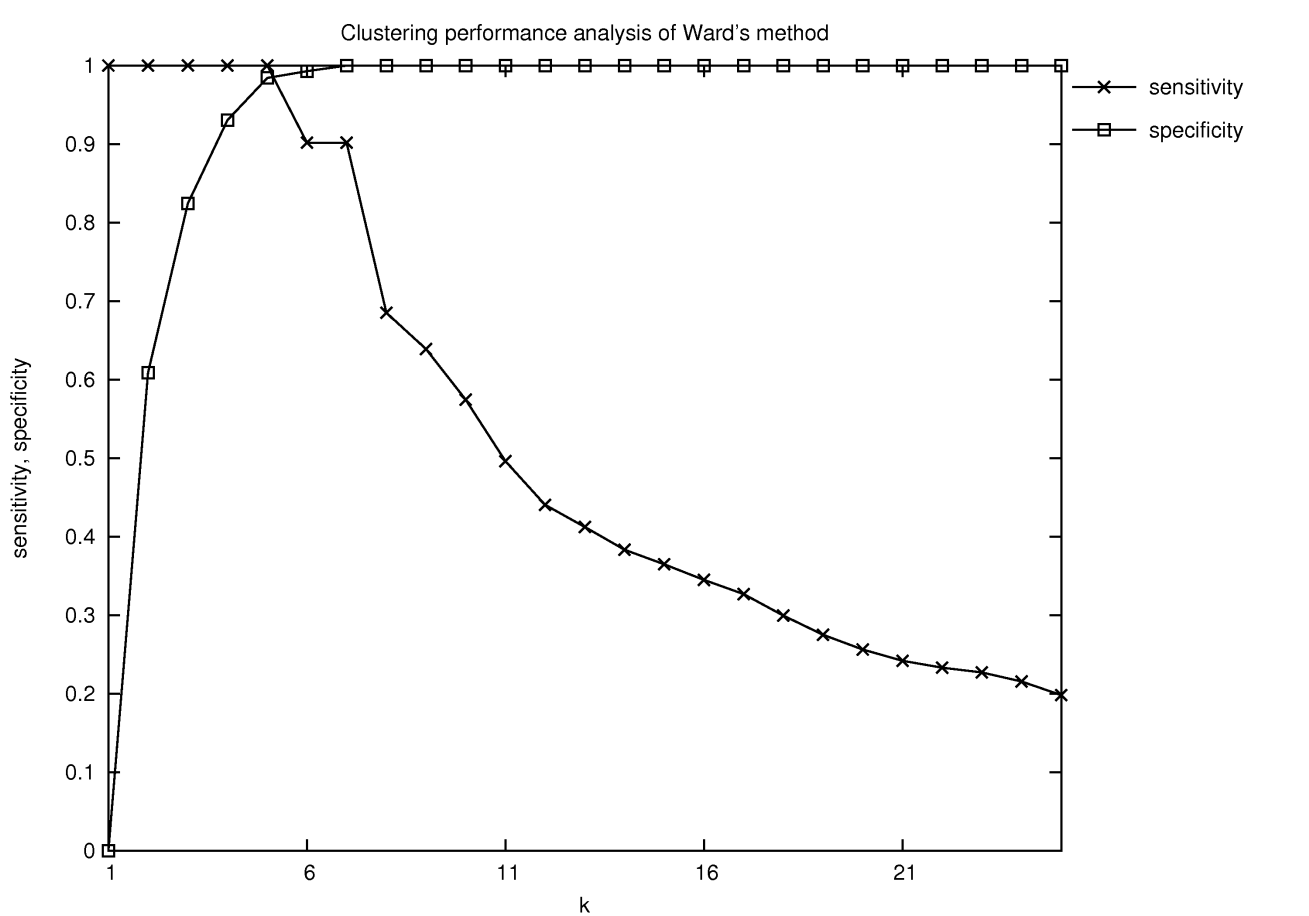
**Quality of the clusterings obtained by Ward's method for each possible level *k***. According to the Kelley-index, the best level is 9. The Kelley-index does not find the best level in the hierarchy since both, specificity and sensitivity, are even higher on level 5.

Clustering the 157 molecules by Ward's method and using the Kelley-index for selecting the best level resulted in nine clusters, i.e., *k *= 9, instead of the expected six. This clustering results in a specificity value of 1.0 and a sensitivity value of 0.6389. Actually, in this case the Kelley index did not choose the optimal level with respect to the reference clustering: In order to exclude the potential bias of the Kelley-index, the sensitivity and specificity values were computed for all possible levels of the clustering hierarchy (Fig. 12). Surprisingly, the fifth level with sensitivity 1 and specificity 0.9842 shows the optimal combination of both values, and not the nineth level as predicted by the Kelley-indexs. Note that none of the levels shows the correct clustering.

**Figure 13:**
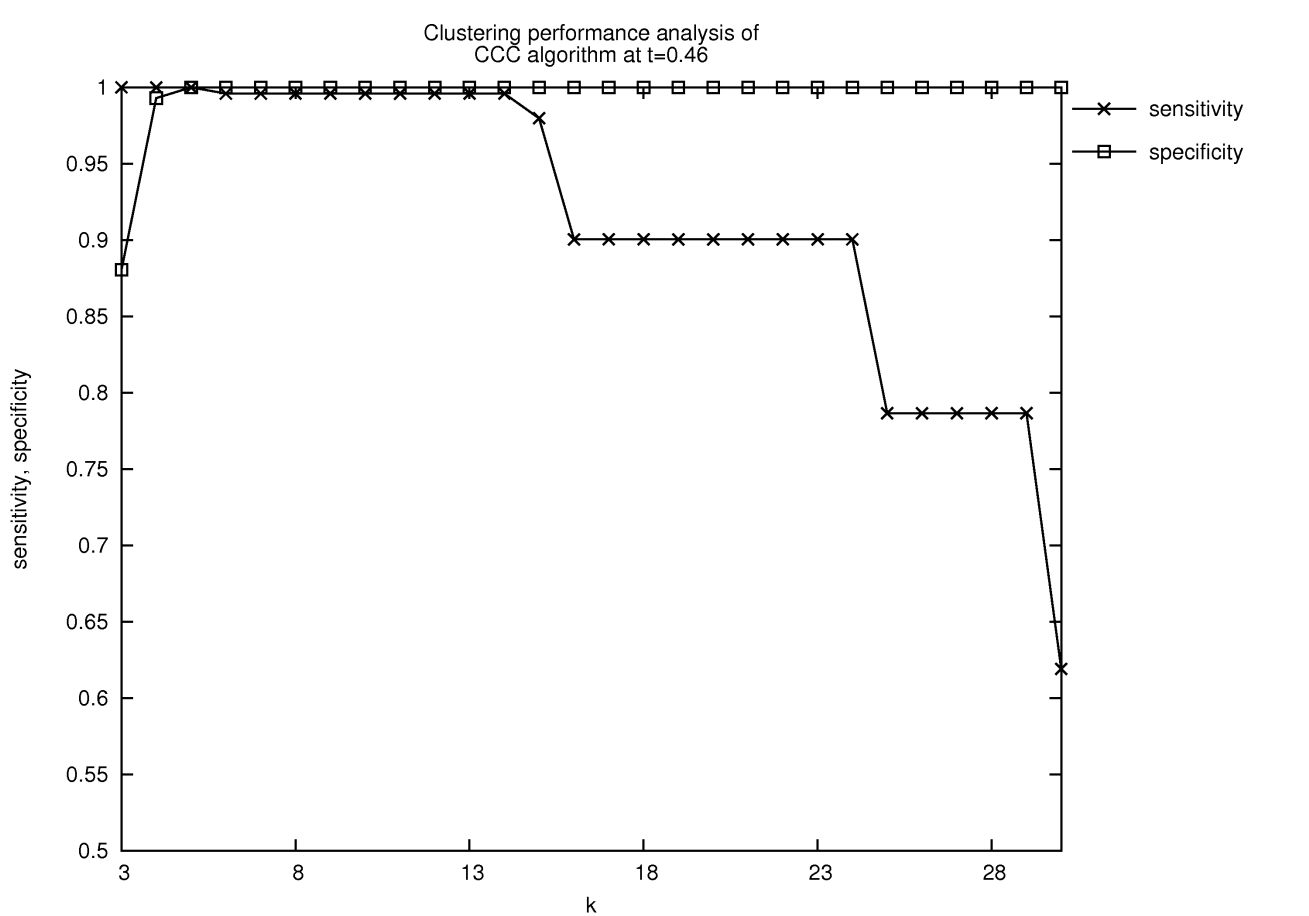
**Quality of the clustering results of CCC at different levels *k *for the similarity threshold *t *= 0.46**. Only for *k *= 5 the same clustering as the reference clustering is computed. It implies that using the CCC algorithm it would be possible to obtain the same clustering as the reference clustering. However, that would require the selection of one specific, i.e., the optimal level of *k*. But without prior knowledge of the reference clustering it is not possible to determine this optimal level.

The CCC performs better in that it at least produces some level in which the six wanted clusters are found: As in Ward's algorithm, we tested the sensitivity and specificity of the resulting clusters at each level of *k*. Here, the situation is a bit better since for *k *= 5 the computed clustering is the same as the reference clustering (Fig. 13). However, without the reference clustering set it would of course not be possible to find the best *k*.

Only LInCS manages to choose from all of the 138 *k*-clique-communities provided by the CCC those six clusters that are the same as the reference clusters in one step by determining the set of maximally cohesive *k*-clique communities (s. *'Finding cohesive k-clique communities: LInCS'*).

**Figure 14:**
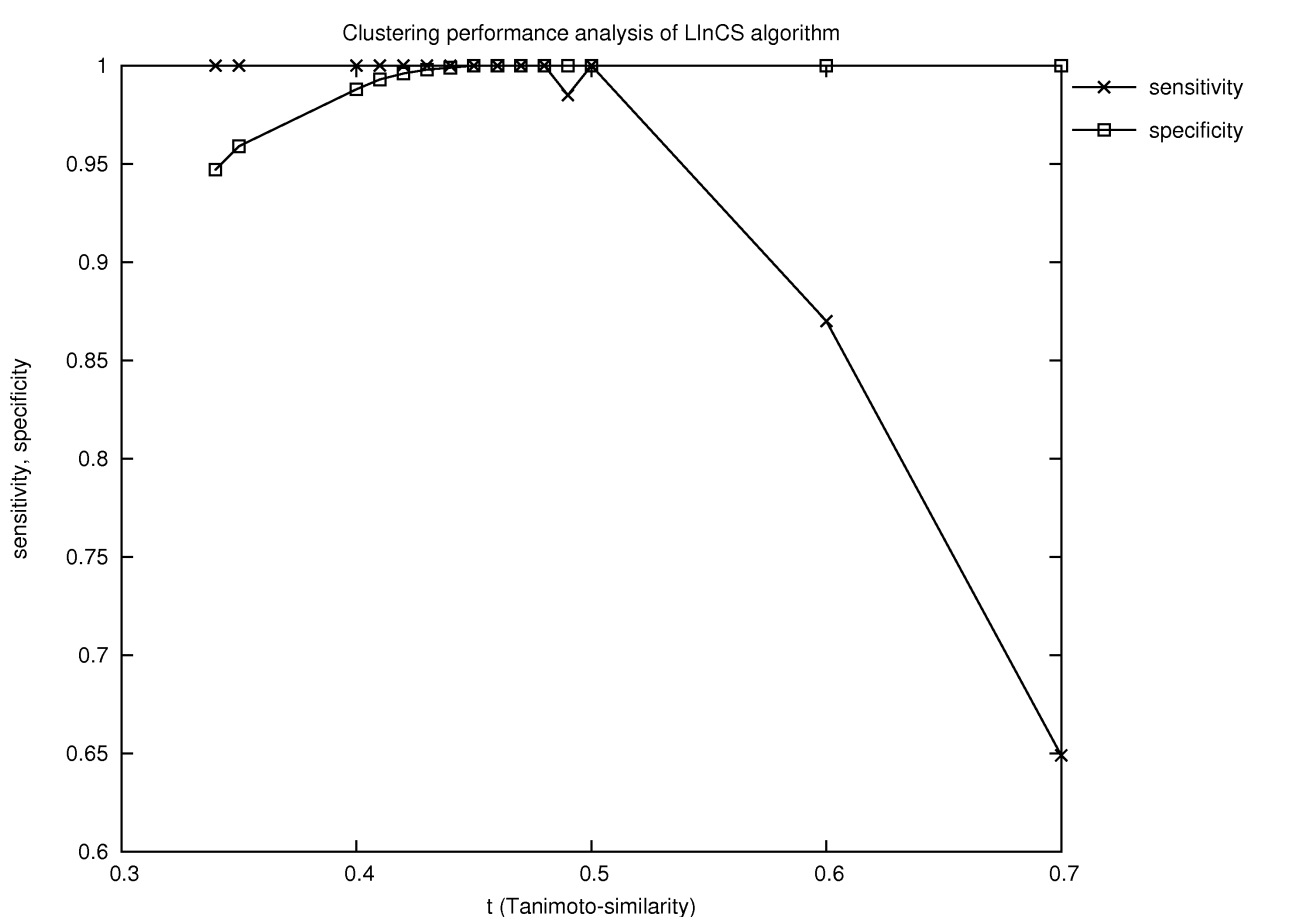
**Clustering results of LInCS**. The figure shows the clustering results of the generated similarity networks at certain similarity threshold values *t*.

Note that these clusters came from different *k *levels although they could also have come from the same level. In any case, this confirms the ability of our LInCS algorithm to resolve the *k*-level selection problem by choosing the maximally cohesive clusters from different levels. It should be noted that the similarity network itself consists of four components, i.e., the six clusters are not directly mapped to six different connected components which would then be easy to find. This argues for the fact that the six resultant clusters were not produced by applying the similarity threshold to the full graph but were produced by LInCS. Both, *sensitivity *and *specificity *reach their maximal value of 1.0 (Fig. 14) at the previously determined optimal similarity threshold at *t *= 0.46. Note also that the clusters are non-overlapping for the chosen similarity threshold of *t *= 0.46. However, generating the similarity network by applying lower threshold values *t*, for instance *t *= 0.40, some of the resultant LInCS clusters are overlapping. This is reasonable considering that the criterion of two molecules to be similar is weakened by decreasing the threshold value. Another consequence is that the sensitivity and specificity values are slightly weaker but still close to their maximal value of 1 as shown in Fig. 14. Note that the correct clustering can be found for *t *in the interval of 0.45-0.48.

LInCS was valuable in finding all six groups of molecules that had the highest similarity as defined by the six combinatorial libraries. We will now show its performance on a data set where no such reference clustering exists but where the homogeneity of the clusters can still be determined.

### Analysis of yeast core protein-protein interaction network

While designing the LInCS algorithm the most important objective was to provide a general method for finding cohesive groups in networks. In order to demonstrate the specific feature of LInCS we applied it to the protein-protein interaction network of *Saccharomyces cerevisiae*. CCC and the consecutive LInCS algorithm can be applied to this network without any transformation and the use of any parameters. The performance of the clustering was measured by computing the sensitivity and specificity values for the biological properties assigned to the proteins as described in *'Performance measurement of clustering molecules'*.

Note that most of the proteins in the data set will not be contained in any of the *k*-clique communities on each possible level *k *(s. Table 3). Thus, all proteins not contained in any community have to be added as singletons to guarantee that each protein is contained in the clustering. We call the percentage of proteins contained in at least one community the *coverage *of a clustering.

**Table 3:** Shown is the number of clusters on each level as computed by the CCC, the coverage of each level, and the total number of clusters, i.e., the sum of the first column and the number of singletons.

**Level**	**Number of clusters of size ≥ 2**	**Coverage**	**Total number of clusters**
3	167	48.6	1523
4	82	23.1	2113
5	48	12.2	2365
6	18	6.0	2502
7	8	3.0	2570
8	4	1.5	2605
9	2	0.07	2623

LInCS	219	33.3	1979

The last line shows the according values for the clustering found by LInCS.

It is clear that from the coverage, the *k *= 3 level would be most reasonable because here 1, 284 out of the 2, 640 proteins are contained in at least one *k*-clique community. On the other hand, the clusters are not all very specific: the minimal specificity of all clusters regarding categories from biological processes, molecular functions or the cellular component is 0.8 in all three cases. Vice versa, the clusterings on higher levels are much more specific but they cover only a few proteins out of all: the minimal specificity of any cluster regarding categories from biological processes is 0.98, for molecular function categories it is 0.99, and for cellular component categories it is 0.99. In essence, the only reasonable levels seem to be located at *k *= 3 or 4. In their paper, Palla et al. picked the *k *= 4 level as the most biologically reasonable by manually looking at the resultant clusterings [11]. According to the following measures the clustering computed by LInCS lies between the two levels: it covers 880 proteins instead of only 609 in level 4 but it is already almost perfectly specific: for all clusters the minimal specificity concerning biological process categories is 0.99, for all molecular function categories it is 1.0 and for all cellular component categories it is 0.99.

**Figure 15:**
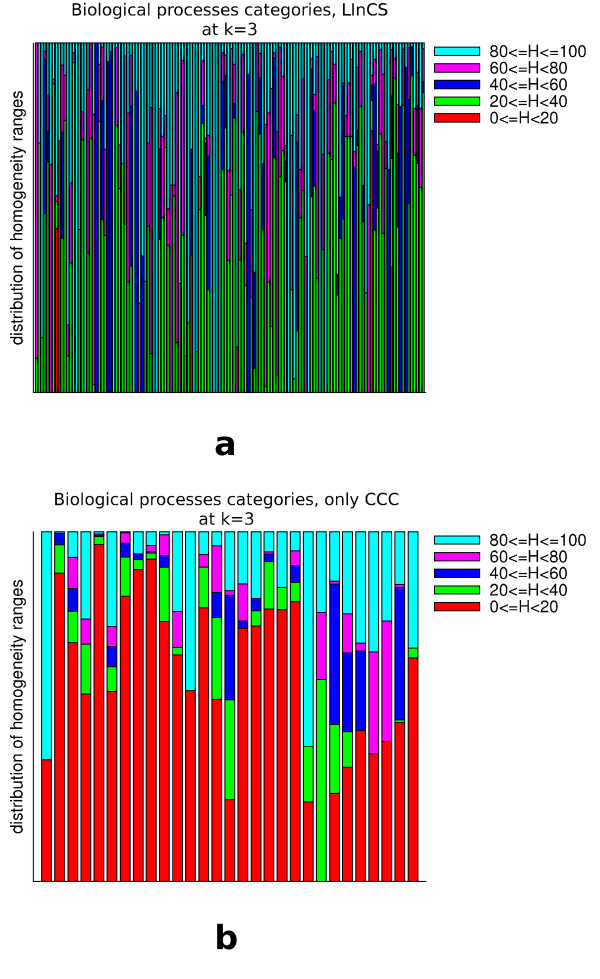
**Shown are for all clusters at level 3 of the CCC the homogeneity value distribution of those biological process categories that are assigned to at least one protein in the cluster**. The upper diagram shows the distributions of those clusters that are included in the LInCS clustering and the lower one those of the clusters that were not included.

**Figure 16:**
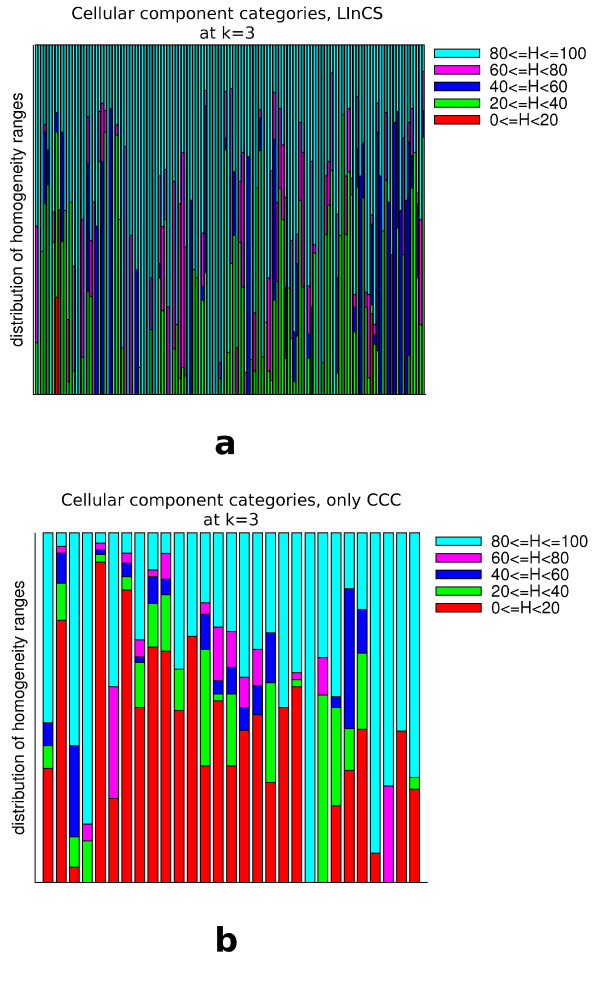
**Shown are for all clusters at level 3 of the CCC the homogeneity value distribution of those cellular component categories that are assigned to at least one protein in the cluster**. The upper diagram shows the distributions of those clusters that are included in the LInCS clustering and the lower one those of the clusters that were not included.

**Figure 17:**
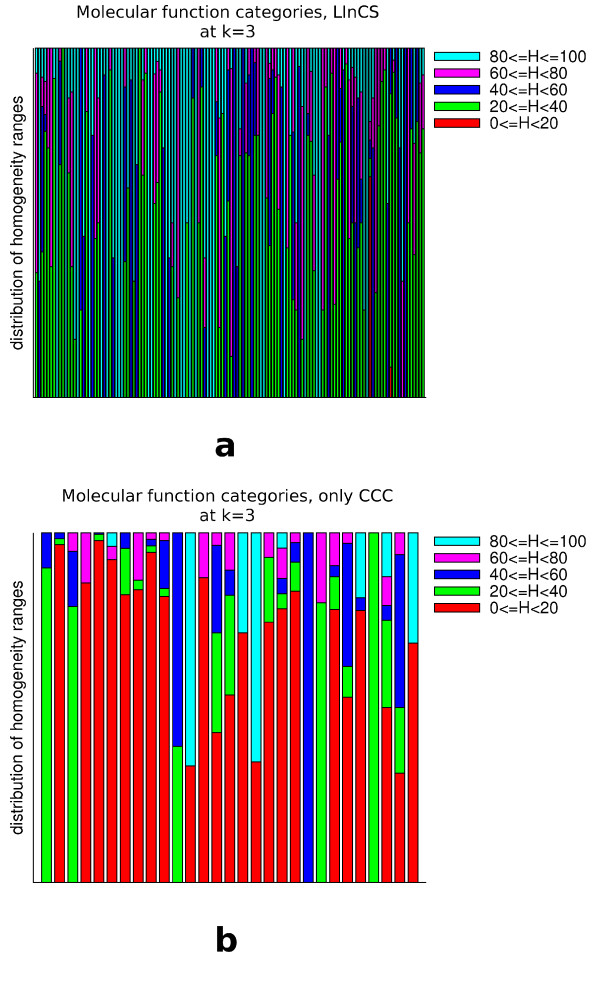
**Shown are for all clusters at level 3 of the CCC the homogeneity value distribution of those molecular function categories that are assigned to at least one protein in the cluster**. The upper diagram shows the distributions of those clusters that are included in the LInCS clustering and the lower one those of the clusters that were not included.

But is the LInCS clustering really preferable over the CCC clustering at level 3 if it covers less proteins? As stated above, the main motivation for the cluster selection algorithm was to find at least one cluster for each protein that is *cohesive*. Of course, the idea is that a cohesive cluster at the graph theoretic level also represents a set of data that is similar to each other. If no such cluster can be found for a given protein it might be better not to include it in an incohesive cluster but rather add it as a singleton. To test whether our notion of cohesiveness really manages to identify the clusters that contain biologically similar proteins we measured the homogeneity of categories in the following way: We define the homogeneity *H*(*C*, *k*, *i*) of cluster i and category *C *at level k as the percentage of proteins assigned to category *C*. For all categories to which at least one protein of the cluster is assigned we computed its homogeneity. Fig. 15, 16 and 17 shows histograms that represent for each (non-singleton) cluster how many categories had a homogeneity of at most 20%, between 20 - 40%, 40 - 60%, 60 - 80% and 80 - 100%. It can be clearly seen that LInCS almost exclusively only picks those clusters in which the homogeneity of the categories is at least between 20% and 40%. Furthermore, those that were not included contain many proteins assigned to categories with a very low homegeneity value. This implies that those clusters that were included to the LInCS clustering by the graph theoretic notion of cohesiveness are much more homogeneous on the biological level than those that were not included. This result strengthens our choice of a cohesiveness measure, but of course, for other data sets other measures of cohesiveness might be appropriate as we will discuss in section *'Generalization of the approach'*.

In summary it turns out that LInCS, by picking clusters from different levels, interpolates between the advantages and disadvantages of level 3 and 4. A closer look at those clusters that were **not **included in LInCS' clustering revealed that they are biologically not very meaningful because the contained proteins do not agree in their biological role. This indicates that the graph theoretic based notion of cohesiveness captures a notion that is also biologically meaningful. Note that LInCS, like the Kelley index for data sets with a similarity measure, does not need any further parameters: Following the paper by Palla et al., different protein-protein interaction networks have been analyzed based on the CCC [11,14,15]. Interestingly, none of the latter publications offered a systematic and general method for extracting one single (overlapping) clustering from the different levels. As shown above, selecting the *k*-clusters by LInCS did not require any parameter or prior knowledge from the user. The experiments confirm that our approach is not only able to produce a cohesive clustering but that the resulting clustering is reasonable for the two data sets we analyzed.

**Figure 18:**
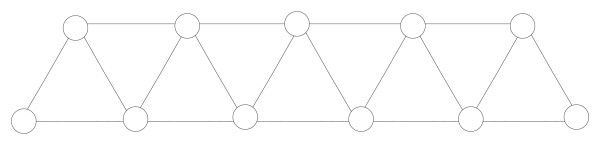
**This exemplary graph will yield a LInCS clustering consisting of only singletons since the one and only 3-clique community consisting of the whole graph is not cohesive**.

As already seen above, LInCS can produce clusterings that put many nodes in singletons. In extreme cases it is possible that CCC produces only non-cohesive *k*-clique communities such that LInCS will produce a clustering in which all nodes are contained as singletons (s. Fig. 18). It is certainly necessary to analyze a data set more closely, as we have done it for the PPI data set above, to see whether this is really a feature of the data set or an artifact of the cohesiveness measure. We will thus now discuss how the idea of finding cohesive clusters can be generalized.

## Generalization of the approach

The idea of choosing maximally cohesive clusters can actually be extended to all hierarchical clusterings to avoid choosing one optimal *level *instead of choosing from all the clusters on all levels at the same time. Of course, for other algorithms and data sets the notion of *cohesiveness *has to be replaced reasonably. To generalize our idea, the chosen *cohesiveness measure *should have the following property: If a cluster on level *k *is *cohesive *this implies that all of its subsets are *cohesive*. Thus, coming from the levels with the lowest *k *the *maximally cohesive clusters *are found first, i.e., in a so-called *divisive clustering algorithm *that starts with the full set and refines this iteratively the algorithm would stop the refinement at a given cluster the moment it is *cohesive*.

## Conclusion

In this article we present a new kind of cluster selection method for hierarchical clusterings, introduced on the example of the *CCC *method by Palla et al. [11]. This method is especially suitable for biological and chemical data sets where no full similarity measure can be defined and in which multiple membership of items to clusters is expected. For these data sets, which can be represented by a graph, we propose to use the *CCC *method and our level-independent cluster selection mechanism *LInCS*. We showed on two examples that this method yields biologically or chemically meaningful clusterings. The method is deterministic, the runtime is quadratic in the number of maximal cliques in the data set and linear in the size of the maximum clique. Under the reasonable assumption that both parameters are small in real-world data sets the runtime is feasible in practice. LInCS uses a graph-theory based and deterministic procedure to find so-called *cohesive clusters *which does not require prior knowledge and expectation on the number and size of the clusters. Although the proposed cohesiveness measure was very successful for the analyzed data sets, other data sets might require other kinds of measures. We thus sketched the generally necessary properties of any kind of cohesiveness measure to adapt our method to other kind of hierarchical clustering methods and data sets. Further research on this topic will have to show whether the level independent method of cluster selection is in general more successful than the classic level selection methods.

## Competing interests

The authors declare that they have no competing interests.

## Authors' contributions

LAZ, GYK and GZK designed the algorithm, proved its correctness, and LAZ implemented it. KAZ and GZK designed the experiments, GZK performed them, KAZ generalized the idea and wrote most of the text. All authors contributed to the text, read and approved the final manuscript.

## Appendix

Proof of Theorem 8

**PROOF**: Let *C *be any cohesive *k*-clique community with *k *≥ 3. As stated by Theorem 4, it might be the superset of one or more *k *+ 1-clique communities. It is easy to see that the *k *+ 1-clique communities contained in it will also be cohesive since any subset of a cohesive set of cliques is also cohesive. Thus, a cohesive superset ensures that all its contained *j*-clique communities with *j *>*k *are also cohesive. Since it is as least as large as those, we do not have to check these *j*-clique communities since they cannot be *maximally cohesive*. On the other hand, the *k*-clique community *C *is itself a subset of exactly one *k *- 1-clique community on level *k *- 1. Thus, if this community is cohesive, *C *is not maximally cohesive. It is now easy to see that by trying for smallest *k *first, and checking all *k*-clique communities on that level for cohesiveness, any cohesive *k*-clique community is at the same time *maximally cohesive*. Thus, all its maximal cliques can be removed from the clique-clique-overlap matrix, thereby reducing the amount of search time on higher levels.

To achieve the runtime, the clique-clique overlap matrix is viewed as an adjacency matrix that builds a graph with at most *γ *nodes and at most *γ*^2 ^edges. Set *k *to 3 and let every entry greater than or equal to *k *- 1 = 2 in the matrix constitute one edge. Computing this graph *G*(*k*) = *G*(3) costs *O*(*γ*^2^). A simple component analysis, e.g., by a breadth first search analysis, yields all 3-clique communities. These can then be checked for cohesiveness and due to the arguments above, any cohesive 3-clique community will at the same time be maximally cohesive. We can now equate a cohesive *k*-clique community with the component in the graph and define a component to be cohesive if the corresponding *k*-clique community is cohesive. All cohesive components can now be deleted from the graph because of the above given argument that they are maximally cohesive. After all components have been checked and while the graph is still non-empty, *k *is increased by one. Then a new graph *G*(*k*) is built where again an edge is drawn between two cliques if their overlap is at least *k *-1. Then, the components in *G*(*k*) are computed and checked for cohesiveness. This cycle of increasing k, building *G*(*k*), the component and cohesiveness analysis and deleting the cohesive components from the graph is then repeated until the graph *G*(*k*) is empty. Note that the graph must eventually be empty since the highest entry in the clique-clique overlap matrix is ≤ *k*_*max*_.

Since a component analysis can be done in linear time in the number of edges in a graph, i.e., in *O*(*γ*^2^), and there are at most *k*_*max *_rounds, this amounts to *O*(*k*_*max*_*γ*^2^). Checking for cohesiveness costs the same as argued above, and thus the total runtime is in *O*(*k*_*max*_*γ*^2^). See Algorithm for a pseudo-code description of LInCS.   ■
